# Protein‐based perioperative nutrition interventions for improving muscle mass and functional outcomes following orthopaedic surgery

**DOI:** 10.1113/EP092237

**Published:** 2025-05-05

**Authors:** Oliver C. Witard, Alix K. Hughes, Paul T. Morgan, Mads Larsen, Phillip J. J. Herrod, Bethan E. Phillips, Jugdeep Dhesi

**Affiliations:** ^1^ Centre for Human and Applied Physiological Sciences King's College London London UK; ^2^ Department of Sport and Exercise Sciences, Institute of Sport Manchester Metropolitan University Manchester UK; ^3^ Arla Foods Ingredients Aarhus Denmark; ^4^ Centre of Metabolism, Ageing and Physiology (COMAP), School of Medicine University of Nottingham, Royal Derby Hospital Derby UK; ^5^ Inflammatory Bowel Disease Service (Surgery), Wythenshawe Hospital University Hospitals of Manchester NHS Foundation Trust London UK; ^6^ Department of Ageing and Health Guy's and St Thomas NHS Trust London UK; ^7^ School of Life Course and Population Sciences King's College London London UK

**Keywords:** osteoarthritis, perioperative nutrition, skeletal muscle

## Abstract

This narrative review provides an overview of protein‐based perioperative nutrition interventions for improving muscle mass and functional outcomes in patients undergoing orthopaedic surgery. Globally, the number of joint replacement procedures continues to rise annually, with beneficial outcomes in terms of pain relief and quality of life. However, orthopaedic surgery is associated with a transient decline in skeletal muscle mass, strength and function, with resulting impact on balance and posture, mobility and an increased risk of falls during the perioperative period. Perioperative nutrition interventions targeted at mitigating muscle atrophy, strength loss and reduced function in response to orthopaedic surgery have primarily focused on essential amino acid and protein supplementation. Promising results have been observed in patients undergoing total knee arthroplasty, total hip replacement, surgical treatment of hip fracture and anterior cruciate ligament reconstruction. Preliminary evidence also suggests a role for perioperative β‐hydroxy‐β‐methylbutyrate supplementation in improving muscle mass and function outcomes following orthopaedic surgery. However, translation of findings from experimental studies into clinical practice is required.

## Abstract (highlights)

- **What is the topic of this review?**
  This narrative review is focused on perioperative nutrition interventions to improve muscle mass and functional outcomes in response to orthopaedic surgery.
- **What advances does it highlight?**
  The evidence regarding effective perioperative nutrition interventions to improve muscle mass and functional outcomes relates to essential amino acid and protein supplementation.

## 1 AN INTRODUCTION TO PERIOPERATIVE MEDICINE IN ORTHOPAEDIC SURGICAL SETTINGS

According to the UK National Joint Registry, a total of 147,728 joint replacement procedures were completed by the NHS in 2023 (‘Home–NJR Surgeon and Hospital Profile’, 2024: https://surgeonprofile.njrcentre.org.uk/). Hip replacement procedures (108,558) were most commonplace, undertaken most often in people aged over 65 years with osteoarthritis. Indeed, based on the ageing population in the UK and across the globe (Global Nutrition Target Collaborators, 2025) and given that arthritis is a disease more prevalent in older adults (Long et al., 2022), the number of joint replacement surgeries in older adults is likely to rise in future years. For example, in the United States, 850,000 total hip and 1.9 million total knee arthroplasty surgeries are predicted in 2030 (Singh et al., 2019), and in the UK, approximately 131,000 total hip and 137,000 total knee replacement surgeries are predicted in 2060 (Matharu et al., 2022). According to recent estimates from the United States, the median total healthcare expenditure calculated at 1 year post total hip or knee joint replacement surgery was $130,314 in robust older adults, increasing to $247,503 in frail patients (Ron et al., 2025).

Multiple clinical trials have reported clinically relevant improvements in pain relief and quality of life following primary hip replacement surgery, irrespective of patient age (Ayers et al., 2022; Bamman et al., 2015; Ninomiya et al., 2020; Singh et al., 2021; Yoshiko et al., 2020). However, the post‐surgical period following orthopaedic surgery can also be associated with impairments in muscle mass, strength and function, leading to instability during walking, abnormal gait patterns, an increased risk of falls and reduced physical activity patterns (Ninomiya et al., 2020). Indeed, orthopaedic surgeries such as anterior cruciate ligament (ACL) reconstruction, bone fractures and joint replacements are associated with substantial and clinically relevant declines in skeletal muscle mass, strength and function over the perioperative period (Atherton et al., 2016; Hirsch et al., 2021; Kramer et al., 2019). In some instances, a compromised muscle functional capacity remains for several years after surgery (Ninomiya et al., 2020; Yoshiko et al., 2020), which presents a precarious situation for older adults, many of whom already face the challenge of age‐related declines in muscle mass and function (Cruz‐Jentoft et al., 2019).

Mechanistically, it has been proposed that the direct stress response to surgery per se leads to a physiological state of whole‐body protein catabolism to accommodate the increased amino acid demand for wound healing, as mediated by a complex cascade of inflammatory, immunological and metabolic events (Gillis & Carli, 2015). Depending upon the degree of surgical insult, this catabolic response can last for a few hours to several days and in some cases up to 2–4 weeks (Hill et al., 1993). Moreover, transient periods of reduced physical activity and/or complete muscle disuse in the lead up to or in the aftermath of surgery may compound the catabolic response to surgery, thereby exacerbating muscle atrophy, strength loss and impaired functional capacity (Nunes et al., 2022; Wall et al., 2013). For instance, a 3–5% decline in vastus lateralis and rectus femoris volume (as measured by magnetic resonance imaging; MRI) was reported 5 months following elective ACL reconstruction surgery in young (∼27 years) patients (Norte et al., 2018). A higher rate of muscle atrophy and strength loss post‐surgery is commonly sustained in older adults (Hvid et al., 2018), with a 14% decline in quadriceps muscle volume reported 2 weeks following total knee arthroplasty in older (> 65 years) patients (Dreyer et al., 2013). This observation may relate, at least in part, to an inferior muscle functional reserve pre‐surgery in older adults and enhanced muscle disuse atrophy compared with their younger counterparts (Hvid et al., 2018). Moreover, large‐scale prospective studies indicate that older adults experience a 5–6% reduction in lean body mass up to 1 year following hip fracture (Fox et al., 2000; Karlsson et al., 1996). Hence, an important and perhaps underappreciated feature of perioperative care relates to the preservation of muscle mass and function in orthopaedic surgery patients. Currently, this notion is especially pertinent in the UK as our healthcare system continues to recover after COVID‐19, with hip and knee replacement procedures still not returned to pre‐pandemic levels as of 2023 (Wainwright et al., 2024).

The developing field of Perioperative Medicine aims to define and implement best practice therapeutic strategies before, during and/or after surgery to improve postoperative outcomes (de Las Casas et al., 2021). Traditional ‘Enhanced Recovery after Surgery (ERAS)’ initiatives have focused on immediate preoperative (with 24 hours of surgery), intraoperative and early postoperative (days after surgery) interventions (Wainwright et al., 2020). The most recent ERAS consensus statement for perioperative care in hip and knee replacement surgery frames nutrition within two contexts (Wainwright et al., 2020). First, the expert group discusses the potential role of preoperative carbohydrate supplementation but does not recommend the implementation of this strategy based on current evidence. Second, it is highlighted that no recommendations can be made regarding postoperative nutritional care as no studies have investigated the impact of feeding or supplementation on discharge criteria. Hence, the current narrative review focuses attention on the evidence base around protein nutrition interventions in order to highlight this option, as well as shed light on nutritional interventions that still require scientific evidence before this strategy might be considered as an ERAS initiative. In contrast, ‘prehabilitation programmes’ should be initiated as soon as a patient is listed for a surgical intervention (Wall et al., 2023; Weimann et al., 2017). Another model of care for older surgical patients also incorporates a medical focus on co‐existing comorbidities and geriatric syndromes such as frailty and cognitive impairment through using Comprehensive Geriatric Assessment and Optimisation Methodology. Nonetheless, all such perioperative approaches aim to provide a framework to facilitate patient return to a normal metabolic state and expedite recovery and have been applied to orthopaedic elective surgery (Lloyd et al., 2024).

Perioperative strategies have also been shown to be effective in reducing length of hospital stay and healthcare costs and have improved time to pre‐surgery levels of physical fitness, mobility and independence. For instance, a comprehensive systematic review recently evaluated the effectiveness of preoperative resistance exercise training in total knee replacement patients, demonstrating a benefit on post‐operative knee function and range of motion in the early (1–3 months) period (Wu et al., 2022). However, orthopaedic surgery often requires a period of partial or complete immobility; hence, in practice, exercise training may not always be viable as a pre‐ or rehabilitation strategy in orthopaedic patient groups, and other interventions should be investigated. Nutrition interventions, albeit most widely established within the context of perioperative parenteral nutrition (Grimes et al., 1987), have also been reported to reduce the length of hospital stay and experiences of severe complications following major orthopaedic surgery (Briguglio & Wainwright, 2025). Current standard clinical nutrition practices for surgery primarily focus on screening and prevention of malnutrition, pre‐surgical fasting protocols and combating post‐surgical insulin resistance (Hirsch et al., 2021), leaving recommendations regarding personalised oral macronutrient composition and timing in relation to surgery less well established (Briguglio & Wainwright, 2022, 2025; Briguglio et al., 2023). Therefore, the over‐arching aim of this narrative review is to discuss the efficacy of protein‐based perioperative nutritional interventions, with a focus on oral feeding rather than parenteral nutrition, to mitigate the decline in muscle mass, strength and function following orthopaedic surgery, with context‐specific applications (i.e., due to sport or fall‐related bone fractures, joint replacements in osteoarthritic patients, etc.) across the life course.

## 2 PERIOPERATIVE NUTRITION FOR ORTHOPAEDIC SURGERY

**FIGURE 1:**
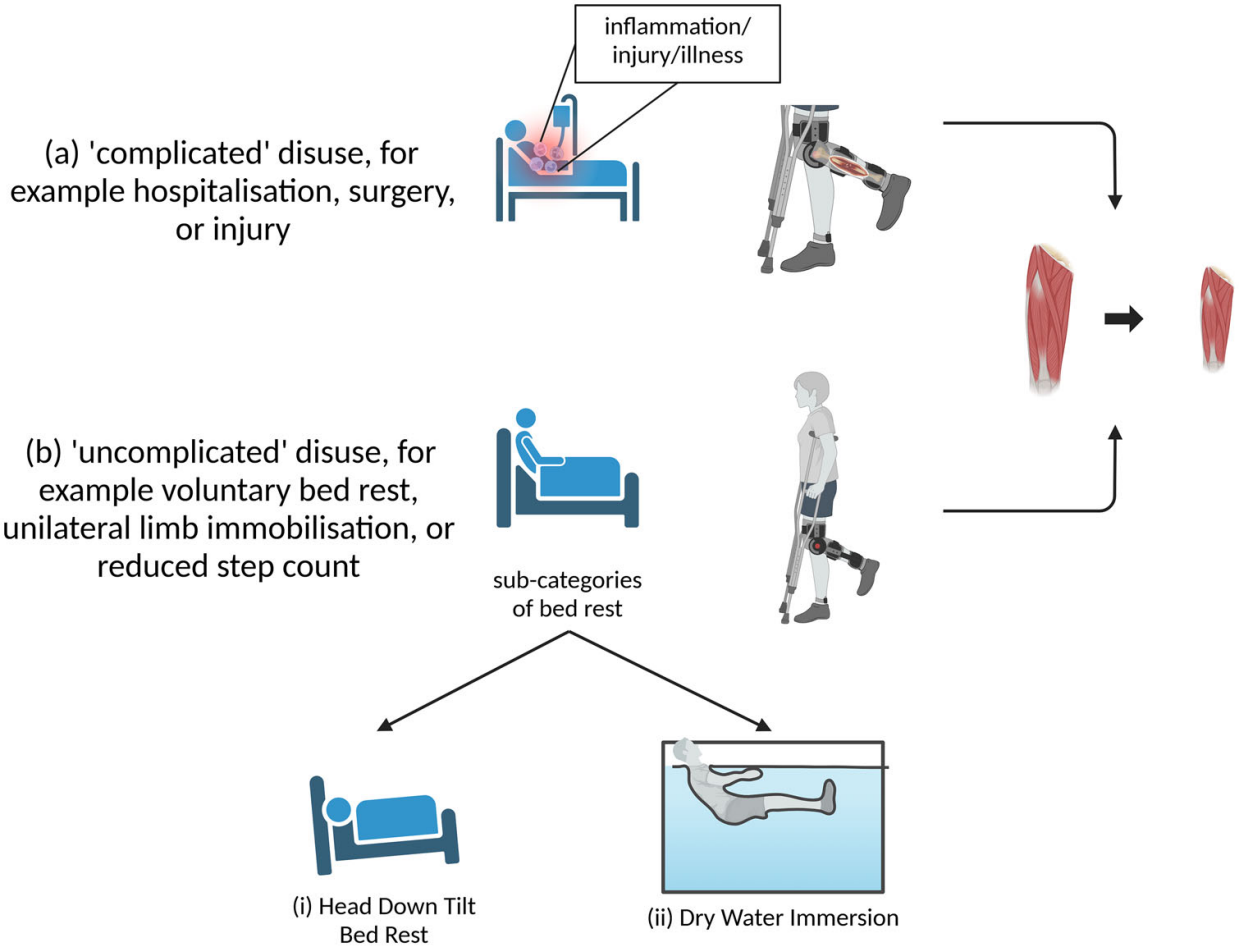
Different disuse scenarios. Complicated disuse refers to periods of disuse due to injury, hospitalization, or diseased states. Uncomplicated disuse refers to periods of disuse due to voluntary bed rest or inactivity, without a prior disease or injury state. Image created with BioRender.com.

The efficacy of nutritional interventions to mitigate the muscle atrophy response to orthopaedic surgery has primarily been extrapolated from proof‐of‐concept experimental studies conducted using simple/uncomplicated models of muscle disuse (i.e., not confounded by an underlying illness) in healthy volunteers (Deane et al., 2024), rather than complicated models of disuse in patient groups undergoing orthopaedic surgery (Figure 1). In brief, randomised controlled trials (RCT) conducted using a simple/uncomplicated disuse model have revealed a lack of clear evidence regarding the benefit of nutritional interventions and most commonly protein or amino acid ingestion in mitigating muscle atrophy in response to muscle disuse in healthy volunteers (for a recent review refer to Hughes et al., 2024). Notwithstanding, clinical trials investigating the efficacy of oral (as opposed to parenteral) perioperative nutrition strategies to mitigate muscle atrophy in response to orthopaedic surgery in patient groups have primarily focussed on protein or amino acid based perioperative interventions, as reviewed in Hirsch et al. (2021). The scientific basis for these trials is underpinned by elaborate metabolic studies in patient groups scheduled for hip (Church et al., 2021) or ACL reconstruction (Howard et al., 2022) surgery that conducted acute measurements of muscle protein turnover, rather than chronic changes in muscle mass, strength and function. Hence, translation to clinically relevant outcomes should be considered with caution. Nonetheless, using a proof‐of‐concept RCT, the administration of a pre‐operative infusion of amino acids was effective in stimulating muscle protein synthesis (MPS) rates and attenuating muscle protein breakdown (MPB) rates during the surgical period in a cohort of middle‐aged elective hip arthroplasty patients (Church et al., 2021). Accordingly, net muscle protein balance (aggregate difference between MPS and MPB over a given time period) was improved and the muscle catabolic response to hip replacement surgery was attenuated in the patient group that received the preoperative amino acid infusion during surgery compared with a standard care group (Church et al., 2021). These data are consistent with clinical trials in which the interoperative and postoperative infusion of amino acids has been shown to increase net muscle protein balance in elective colorectal surgery patients (Donatelli, Schricker, Mistraletti et al., 2006, Donatelli, Schricker, Parrella et al., 2006). However, it should be noted that no control group was included in either study (Donatelli, Schricker, Mistraletti et al., 2006, Donatelli, Schricker, Parrella et al., 2006), and hence it is difficult to draw firm conclusions regarding the impact of the amino acid infusion. Moreover, it is conceivable that the intravenous infusion of amino acids compared to oral ingestion of amino acids may elicit divergent physiological responses. For example, amino acid infusions elicit more rapid and pronounced plasma amino acid concentrations, whilst oral amino acid ingestion elicits a greater postprandial insulinaemia (Abdulla et al., 2019). Hence, whilst these preliminary studies show promise in terms of a perioperative nutrition strategy, translation to real world application of supplemental or dietary interventions remains unclear.

The second metabolic study is a pilot RCT that manipulated dietary protein intake (1.2 vs. 1.9 g kgBM^−1^ day^−1^) during a 2‐week period of muscle disuse prior to primary ACL reconstruction in a small cohort (*n* = 3 protein, *n* = 3 control) of young men (Howard et al., 2022). MPS rates were increased in the high protein diet group over this preoperative period, indicating a potential benefit of increasing dietary protein intake during inevitable periods of complete muscle disuse or markedly reduced levels of physical activity prior to orthopaedic surgery. To this end, no metabolic studies have investigated the muscle anabolic response to oral (rather than parenteral) protein or amino acid feeding during the post‐operative rehabilitation period within the context of orthopaedic surgery. In addition, limited conclusions can be drawn from these studies (and indeed studies in uncomplicated models of disuse) since they do not capture the entire perioperative period, inclusive of pre‐, during and post‐operative periods, or combine acute metabolic readouts with clinically relevant measurements of muscle function. As such, this area warrants further investigation to enhance our understanding of the metabolic cascades underpinning the entire surgical process. Nevertheless, these data provide the scientific rationale to undertake large‐scale RCTs into oral protein‐based feeding strategies to mitigate decrements in muscle mass, strength and function in the context of orthopaedic surgery.

## 3 ESSENTIAL AMINO ACID SUPPLEMENTATION

Longitudinal studies into the efficacy of essential amino acid (EAA) supplementation as a perioperative nutrition strategy have generally reported favourable outcomes with regards to mitigating post‐surgical declines in skeletal muscle mass, strength and/or function in orthopaedic patient groups (George et al., 2023; Reider et al., 2023). A series of clinical trials have been conducted at the University of Oregon in this area (Dreyer et al., 2018, 2013; Muyskens et al., 2019, 2020). These studies utilised a randomised, double blinded, placebo‐controlled experimental design and recruited a relatively large cohort of middle‐aged (*n* = 39) and older (*n* = 28) total knee arthroplasty patients to ingest 20 g of EAA (equivalent to ∼40 g protein) or a placebo (non‐essential amino acid (NEAA) mixture or alanine) twice daily for 7 days before and 2 (Dreyer et al., 2013) or 6 (Dreyer et al., 2018) weeks after surgery. This elegant research design included a within‐subjects control study arm with measurements of muscle (quadriceps) volume (using gold standard MRI), strength and function also conducted on the contralateral, non‐operated leg. A consistent finding across studies was that EAA ingestion attenuated the magnitude of muscle atrophy by 5–12% when measured at 2‐ or 6‐weeks post‐surgery, although the preservation of muscle mass with EAA ingestion did not consistently translate to improvements in strength and functional mobility over the 6‐week rehabilitation period (Dreyer et al., 2018, 2013). The absence of an exercise component within the perioperative medicine strategy likely explains, at least in part, the lack of improvements in strength and function. Interestingly, although the degree of muscle atrophy was more substantial during the early versus later post‐surgical period (as has been previously reported; Hardy et al., 2022), the favourable effect of the EAA intervention was comparable between post‐surgery time points. The evidence base regarding the perioperative role of EAA supplementation also extends to RCTs in hip fracture and hip arthroplasty patients, whereby improvements in muscle mass (Hendrickson et al., 2022), strength (Borsheim et al., 2008; Ferrando AA et al., 2013) and function (Aquilani et al., 2019; Baldissarro et al., 2016; Invernizzi et al., 2019) have consistently been reported during the post‐surgical period. Hence, there is accumulating evidence that EAA supplementation represents an intervention that potentially improves outcomes following orthopaedic surgery.

## 4 PROTEIN SUPPLEMENTATION

Guidelines for surgical patients generally recommend an adequate preoperative dietary protein intake in excess of 1.2 g kgBM^−1^ day^−1^ (Wischmeyer et al., 2018), which is substantially higher than the RDA for protein of ∼0.8 g kgBM^−1^ day^−1^. However, it has been suggested that even higher protein intakes may be warranted for individuals during injury scenarios (Tipton, 2010) and studies into optimal protein recommendations for orthopaedic surgery patients are needed (McKendry et al., 2024). Interestingly, protein intakes of <0.6 g kgBM^−1^ day^−1^ have been reported in older patients during a 6‐day period of hospitalisation following total hip or knee arthroplasty (Weijzen et al., 2019) which, although higher than reported for abdominal surgery patients (Hardy et al., 2023), remains lower than deemed adequate. Accordingly, the preponderance of longitudinal studies have investigated the effects of postoperative protein supplementation on more subjective clinical outcomes during rehabilitation, rather than more objective muscle‐specific endpoints in older hip fracture patients (Espaulella et al., 2000; Schurch et al., 1998; Tidermark et al., 2004). For instance, improvements in activities of daily living and quality of life were reported in patients randomised to 20 g/day of protein supplementation (a protein‐rich formula, Fortimel) versus control (standard care) when measured at 6 months post‐hip surgery (Tidermark et al., 2004). Moreover, a separate RCT conducted muscle‐specific measurements in young ACL reconstruction patients and reported that the combined ingestion of protein and carbohydrate (10 g protein from skimmed‐milk and soybeans, 7 g carbohydrate) augmented improvements in muscle mass and strength outcomes following 12 weeks of resistance training rehabilitation (Holm et al., 2006). Hence, the evidence base pertaining to the longer‐term postoperative effect of protein supplementation appears promising. However, it must be acknowledged that studies investigating longer‐term interventions may no longer be impacting the post‐surgical period but rather be investigating the impact of nutritional supplementation on exercise training interventions during rehabilitation. Hence, future studies are warranted to determine the optimal post‐surgical time over which to implement nutritional interventions in the context of rehabilitation. Nonetheless, several RCTs have been conducted investigating protein or amino acid supplementation in orthopaedic patient groups, indicating a beneficial effect on muscle function and quality of life outcomes. As such, these interventions may be worth considering in the first instance for clinicians with the goal of implementing dietary recommendations for orthopaedic patients. Moreover, follow‐up studies are warranted to investigate the effect of perioperative protein supplementation on more sensitive physiological measurements of muscle mass, strength and function in orthopaedic patient groups.

## 5 β‐HYDROXY‐β‐METHYLBUTYRATE

Beyond protein and/or EAA ingestion, another perioperative nutrition strategy in terms of mitigating the decline in muscle mass, strength and function following orthopaedic surgery includes β‐hydroxy‐β‐methylbutyrate (HMB) supplementation (Marshall et al., 2020). HMB is a metabolite of leucine catabolism in the liver (van Koevering & Nissen, 1992) and skeletal muscle that has been shown to stimulate MPS and attenuate MPB rates in reference to in vitro cell culture models (Eley et al., 2007) and in vivo human clinical trials (Wilkinson et al., 2013, 2018). Accordingly, improvements in muscle mass and strength with 2–3 g/day of HMB supplementation have been reported in older adults (Flakoll et al., 2004) and, most notably, an attenuated decline in muscle mass in response to 10 days of bedrest in older adults (Deutz et al., 2013). Moreover, in an RCT, pre‐ and postoperative supplementation with a combination of HMB, arginine and glutamine was shown to expedite the restoration of quadriceps strength in older adult total knee arthroplasty patients (Nishizaki et al., 2015). However, while a recent meta‐analysis that investigated the impact of HMB on a broad range of clinical conditions reported a beneficial effect on muscle mass and strength, the effect sizes were small (Bear et al., 2019). In addition, no studies were considered low risk of bias, and many of the included studies did not investigate HMB in isolation, but rather alongside other supplements. Therefore, while there may be a potential clinical benefit of HMB, it is difficult to delineate if this effect is due to HMB alone. Hence, it would be recommended that further high‐quality clinical trials should be undertaken to further investigate the efficacy of HMB interventions before it is considered by clinicians in the context of perioperative nutrition for orthopaedic patients.

## 6 CONCLUSION

Experimental and clinical studies suggest EAA supplementation is a promising perioperative nutritional strategy to mitigate declines in muscle mass, strength and function following orthopaedic surgery, with some evidence suggesting a role for protein and HMB supplementation. The National Institutes of Health (NIH) National Center for Advancing Translational Sciences defines translational research as ‘the process of turning observations in the laboratory, clinic and community into interventions that improve the health of individuals’ (NCATS Overview, National Center for Advancing Translational Sciences, 2024: https://ncats.nih.gov/about/ncats‐overview/strategic‐plan/overview‐of‐ncats). To explore and translate findings from experimental studies of perioperative nutrition for patients undergoing orthopaedic elective surgery into clinical practice requires careful consideration of several factors. These factors include working with patients and other stakeholders to understand: (i) the prevalence of underlying nutritional and muscle disorders, (ii) acceptability and feasibility of pre‐, intra‐ and postoperative nutritional interventions, and (iii) outcomes that matter to this patient group and the healthcare economy. In addition, there is a need for careful consideration of the research methodologies to be employed that are most likely to lead to timely change in practice. For example, randomised clinical trials and/or implementation‐effectiveness studies and there is a need for evaluation of cost effectiveness of single component and/or multicomponent interventions (e.g., nutrition alone or nutrition and exercise). Accordingly, patient and public involvement and engagement (PPIE) activities and feasibility studies that provide a signal of efficacy and potential for implementation (translation into clinical practice) are crucial first steps in progressing this exciting field of perioperative nutrition in orthopaedic surgery.

## AUTHOR CONTRIBUTIONS

Alix K. Hughes and Oliver C. Witard were responsible for the concept of the review. All authors contributed to the design and writing of the manuscript. All authors approved the final version of the manuscript and agree to be accountable for all aspects of the work in ensuring that questions related to the accuracy or integrity of any part of the work are appropriately investigated and resolved. All persons designated as authors qualify for authorship, and all those who qualify for authorship are listed.

## CONFLICT OF INTEREST

None declared.

## FUNDING INFORMATION

None.

## References

[eph13865-bib-0001] Abdulla, H. , Bass, J. J. , Stokes, T. , Gorissen, S. H. M. , McGlory, C. , Phillips, B. E. , Phillips, S. M. , Smith, K. , Idris, I. , & Atherton, P. J. (2019). The effect of oral essential amino acids on incretin hormone production in youth and ageing. Endocrinology, Diabetes & Metabolism, 2(4), e00085. 10.1002/edm2.85 PMC6775449 31592446

[eph13865-bib-0002] Aquilani, R. , Zuccarelli Ginetto, C. , Rutili, C. , Pisano, P. , Pasini, E. , Baldissarro, E. , Verri, M. , & Boschi, F. (2019). Supplemented amino acids may enhance the walking recovery of elderly subjects after hip fracture surgery. Aging Clinical and Experimental Research, 31(1), 157–160. 29667153 10.1007/s40520-018-0941-x

[eph13865-bib-0003] Atherton, P. J. , Greenhaff, P. L. , Phillips, S. M. , Bodine, S. C. , Adams, C. M. , & Lang, C. H. (2016). Control of skeletal muscle atrophy in response to disuse: Clinical/preclinical contentions and fallacies of evidence. American Journal of Physiology. Endocrinology and Metabolism, 311(3), E594–E604. 27382036 10.1152/ajpendo.00257.2016 PMC5142005

[eph13865-bib-0004] Ayers, D. C. , Yousef, M. , Zheng, H. , Yang, W. , & Franklin, P. D. (2022). Do patient outcomes vary by patient age following primary total hip arthroplasty? Journal of Arthroplasty, 37(7), S510–S516. 35292339 10.1016/j.arth.2022.03.032

[eph13865-bib-0005] Baldissarro, E. , Aquilani, R. , Boschi, F. , Baiardi, P. , Iadarola, P. , Fumagalli, M. , Pasini, E. , Verri, M. , Dossena, M. , Gambino, A. , Cammisuli, S. , & Viglio, S. (2016). The hip functional retrieval after elective surgery may be enhanced by supplementing essential amino acids. BioMed Research International, 2016, 1–10. 10.1155/2016/9318329 PMC4823478 27110573

[eph13865-bib-0006] Bamman, M. M. , Ferrando, A. A. , Evans, R. P. , Stec, M. J. , Kelly, N. A. , Gruenwald, J. M. , Corrick, K. L. , Trump, J. R. , & Singh, J. A. (2015). Muscle inflammation susceptibility: A prognostic index of recovery potential after hip arthroplasty? American Journal of Physiology. Endocrinology and Metabolism, 308(8), E670–E679. 25670829 10.1152/ajpendo.00576.2014 PMC4398830

[eph13865-bib-0007] Bear, D. E. , Langan, A. , Dimidi, E. , Wandrag, L. , Harridge, S. D. R. , Hart, N. , Connolly, B. , & Whelan, K. (2019). β‐Hydroxy‐β‐methylbutyrate and its impact on skeletal muscle mass and physical function in clinical practice: A systematic review and meta‐analysis. American Journal of Clinical Nutrition, 109(4), 1119–1132. 30982854 10.1093/ajcn/nqy373

[eph13865-bib-0008] Borsheim, E. , Bui, Q. T. , Tissier, S. , Kobayashi, H. , Ferrando, A. A. , & Wolfe, R. R. (2008). Effect of amino acid supplementation on muscle mass, strength, and physical function in the elderly. Clinical Nutrition, 27(2), 189–195. 18294740 10.1016/j.clnu.2008.01.001 PMC2430042

[eph13865-bib-0009] Briguglio, M. , & Wainwright, T. W. (2022). Nutritional and physical prehabilitation in elective orthopedic surgery: Rationale and proposal for implementation. Therapeutics and Clinical Risk Management, 18, 21–30. 35023922 10.2147/TCRM.S341953 PMC8747789

[eph13865-bib-0010] Briguglio, M. , & Wainwright, T. W. (2025). Towards personalised nutrition in major orthopaedic surgery: Elements of care process. Nutrients, 17(4), 700. 40005028 10.3390/nu17040700 PMC11858543

[eph13865-bib-0011] Briguglio, M. , Wainwright, T. W. , Southern, K. , Riso, P. , Porrini, M. , & Middleton, R. G. (2023). Healthy eating for elective major orthopedic surgery: Quality, quantity, and timing. Journal of Multidisciplinary Healthcare, 16, 2081–2090. 37521366 10.2147/JMDH.S415116 PMC10377616

[eph13865-bib-0012] Church, D. D. , Schutzler, S. E. , Wolfe, R. R. , & Ferrando, A. A. (2021). Perioperative amino acid infusion reestablishes muscle net balance during total hip arthroplasty. Physiological Reports, 9(18), e15055. 34558214 10.14814/phy2.15055 PMC8461212

[eph13865-bib-0013] Cruz‐Jentoft, A. , Bahat, G. , Bauer, J. , Boirie, Y. , Bruyere, O. , Cederholm, T. , Cooper, C. , Landi, F. , Rolland, Y. , Sayer, A. A. , Schneider, S. M. , Sieber, C. C. , Topinkova, E. , Vandewoude, M. , Visser, M. , Zamboni, M. , & Writing Group for the European Working Group on Sarcopenia in Older People 2 (EWGSOP2), and the Extended Group for EWGSOP2 . (2019). Sarcopenia: Revised European consensus on definition and diagnosis. Age and Ageing, 48(1), 16–31. 30312372 10.1093/ageing/afy169 PMC6322506

[eph13865-bib-0014] Deane, C. S. , Piasecki, M. , & Atherton, P. J. (2024). Skeletal muscle immobilisation‐induced atrophy: Mechanistic insights from human studies. Clinical Science, 138(12), 741–756. 38895777 10.1042/CS20231198 PMC11186857

[eph13865-bib-0015] de Las Casas, R. , Meilak, C. , Whittle, A. , Partridge, J. , Adamek, J. , Sadler, E. , Sevdalis, N. , & Dhesi, J. (2021). Establishing a perioperative medicine for older people undergoing surgery service for general surgical patients at a district general hospital. Clinical Medicine, 21(6), e608–e614. 34862220 10.7861/clinmed.2021-0356 PMC8806285

[eph13865-bib-0016] Deutz, N. E. P. , Pereira, S. L. , Hays, N. P. , Oliver, J. S. , Edens, N. K. , Evans, C. M. , & Wolfe, R. R. (2013). Effect of beta‐hydroxy‐beta‐methylbutyrate (HMB) on lean body mass during 10 days of bed rest in older adults. Clinical Nutrition, 32(5), 704–712. 23514626 10.1016/j.clnu.2013.02.011

[eph13865-bib-0017] Donatelli, F. , Schricker, T. , Mistraletti, G. , Asenjo, F. , Parrella, P. , Wykes, L. , & Carli, F. (2006). Postoperative infusion of amino acids induces a positive protein balance independently of the type of analgesia used. Anesthesiology, 105(2), 253–259. 16871058 10.1097/00000542-200608000-00007

[eph13865-bib-0018] Donatelli, F. , Schricker, T. , Parrella, P. , Asenjo, F. , Wykes, L. , & Carli, F. (2006). Intraoperative infusion of amino acids induces anabolism independent of the type of anesthesia. Anesthesia and Analgesia, 103(6), 1549–1556. 17122238 10.1213/01.ane.0000243332.08397.52

[eph13865-bib-0019] Dreyer, H. C. , Owen, E. C. , Strycker, L. A. , Smolkowski, K. , Muyskens, J. B. , Kirkpatrick, T. K. , Christie, A. D. , Kuehl, K. S. , Lantz, B. A. , Shah, S. N. , Mohler, C. G. , & Jewett, B. A. (2018). Essential amino acid supplementation mitigates muscle atrophy after total knee arthroplasty: A randomized, double‐blind, placebo‐controlled trial. JB&JS Open Access, 3(2), e0006. 30280129 10.2106/JBJS.OA.18.00006 PMC6145559

[eph13865-bib-0020] Dreyer, H. C. , Strycker, L. A. , Senesac, H. A. , Hocker, A. D. , Smolkowski, K. , Shah, S. N. , & Jewett, B. A. (2013). Essential amino acid supplementation in patients following total knee arthroplasty. Journal of Clinical Investigation, 123(11), 4654–4666. 24135139 10.1172/JCI70160 PMC3809795

[eph13865-bib-0021] Eley, H. L. , Russell, S. T. , Baxter, J. H. , Mukerji, P. , & Tisdale, M. J. (2007). Signaling pathways initiated by beta‐hydroxy‐beta‐methylbutyrate to attenuate the depression of protein synthesis in skeletal muscle in response to cachectic stimuli. American Journal of Physiology. Endocrinology and Metabolism, 293(4), E923–E931. 17609254 10.1152/ajpendo.00314.2007

[eph13865-bib-0022] Espaulella, J. , Guyer, H. , Diaz‐Escriu, F. , Mellado‐Navas, J. A. , Castells, M. , & Pladevall, M. (2000). Nutritional supplementation of elderly hip fracture patients. A randomized, double‐blind, placebo‐controlled trial. Age and Ageing, 29(5), 425–431. 11108415 10.1093/ageing/29.5.425

[eph13865-bib-0023] Ferrando, A. A. , Bamman, M. M. , Schutzler, S. E. , Spence, H. J. , Dawson, A. M. , Evans, R. P. , & Wolfe, R. R. (2013). Increased nitrogen intake following hip arthroplasty expedites muscle strength recovery. The Journal of Aging Research & Clinical Practice, 2, 369–75.

[eph13865-bib-0024] Flakoll, P. , Sharp, R. , Baier, S. , Levenhagen, D. , Carr, C. , & Nissen, S. (2004). Effect of beta‐hydroxy‐beta‐methylbutyrate, arginine, and lysine supplementation on strength, functionality, body composition, and protein metabolism in elderly women. Nutrition, 20(5), 445–451. 15105032 10.1016/j.nut.2004.01.009

[eph13865-bib-0025] Fox, K. M. , Magaziner, J. , Hawkes, W. G. , Yu‐Yahiro, J. , Hebel, J. R. , Zimmerman, S. I. , Holder, L. , & Michael, R. (2000). Loss of bone density and lean body mass after hip fracture. Osteoporosis International, 11(1), 31–35. 10663356 10.1007/s001980050003

[eph13865-bib-0026] George, A. , Holderread, B. M. , Lambert, B. S. , Harris, J. D. , & McCulloch, P. C. (2023). Post‐operative protein supplementation following orthopaedic surgery: A systematic review. Sports Medicine and Health Science, 6(1), 16–24. 38463662 10.1016/j.smhs.2023.08.002 PMC10918348

[eph13865-bib-0027] Gillis, C. , & Carli, F. (2015). Promoting perioperative metabolic and nutritional care. Anesthesiology, 123(6), 1455–1472. 26248016 10.1097/ALN.0000000000000795

[eph13865-bib-0028] Global Nutrition Target Collaborators . (2025). Global, regional, and national progress towards the 2030 global nutrition targets and forecasts to 2050: A systematic analysis for the Global Burden of Disease Study 2021. Lancet, 404(10471), 2543–2583. 39667386 10.1016/S0140-6736(24)01821-X PMC11703702

[eph13865-bib-0029] Grimes, C. J. , Younathan, M. T. , & Lee, W. C. (1987). The effect of preoperative total parenteral nutrition on surgery outcomes. Journal of the American Dietetic Association, 87(9), 1202–1206. 3114353

[eph13865-bib-0030] Hardy, E. J. , Deane, C. S. , Lund, J. N. , & Phillips, B. E. (2023). Loss of muscle mass in the immediate post‐operative period is associated with inadequate dietary protein and energy intake. European Journal of Clinical Nutrition, 77(4), 503–505. 36702923 10.1038/s41430-023-01264-0 PMC10115623

[eph13865-bib-0031] Hardy, E. J. O. , Inns, T. B. , Hatt, J. , Doleman, B. , Bass, J. J. , Atherton, P. J. , Lund, J. N. , & Phillips, B. E. (2022). The time course of disuse muscle atrophy of the lower limb in health and disease. Journal of Cachexia, Sarcopenia and Muscle, 13(6), 2616–2629. 36104842 10.1002/jcsm.13067 PMC9745468

[eph13865-bib-0032] Hendrickson, N. R. , Davison, J. , Glass, N. A. , Wilson, E. S. , Miller, A. , Leary, S. , Lorentzen, W. , Karam, M. D. , Hogue, M. , Marsh, J. L. , & Willey, M. C. (2022). Conditionally essential amino acid supplementation reduces postoperative complications and muscle wasting after fracture fixation: A randomized controlled trial. The Journal of Bone and Joint Surgery, 104(9), 759–766. 35286282 10.2106/JBJS.21.01014

[eph13865-bib-0033] Hill, G. L. , Douglas, R. G. , & Schroeder, D. (1993). Metabolic basis for the management of patients undergoing major surgery. World Journal of Surgery, 17(2), 146–153. 8511907 10.1007/BF01658920

[eph13865-bib-0034] Hirsch, K. R. , Wolfe, R. R. , & Ferrando, A. A. (2021). Pre‐ and post‐surgical nutrition for preservation of muscle mass, strength, and functionality following orthopedic surgery. Nutrients, 13(5), 1675. 34063333 10.3390/nu13051675 PMC8156786

[eph13865-bib-0035] Holm, L. , Esmarck, B. , Mizuno, M. , Hansen, H. , Suetta, C. , Holmich, P. , Krogsgaard, M. , & Kjaer, M. (2006). The effect of protein and carbohydrate supplementation on strength training outcome of rehabilitation in ACL patients. Journal of Orthopaedic Research, 24(11), 2114–2123. 16917926 10.1002/jor.20147

[eph13865-bib-0036] Howard, E. E. , Margolis, L. M. , Fussell, M. A. , Rios, C. G. , Meisterling, E. M. , Lena, C. J. , Pasiakos, S. M. , & Rodriguez, N. R. (2022). Effect of high‐protein diets on integrated myofibrillar protein synthesis before anterior cruciate ligament reconstruction: A randomized controlled pilot study. Nutrients, 14(3), 563. 35276922 10.3390/nu14030563 PMC8840691

[eph13865-bib-0037] Hughes, A. K. , Francis, T. , Rooney, J. , Pollock, R. , & Witard, O. C. (2024). The effect of protein or amino acid provision on immobilization‐induced muscle atrophy in healthy adults: A systematic review and meta‐analysis. Experimental Physiology, 109(6), 873–888. 38424716 10.1113/EP090434 PMC11140175

[eph13865-bib-0038] Hvid, L. G. , Aagaard, P. , Ortenblad, N. , Kjaer, M. , & Suetta, C. (2018). Plasticity in central neural drive with short‐term disuse and recovery—Effects on muscle strength and influence of aging. Experimental Gerontology, 106, 145–153. 29476804 10.1016/j.exger.2018.02.019

[eph13865-bib-0039] Invernizzi, M. , de Sire, A. , D'Andrea, F. , Carrera, D. , Reno, F. , Migliaccio, S. , Iolascon, G. , & Cisari, C. (2019). Effects of essential amino acid supplementation and rehabilitation on functioning in hip fracture patients: A pilot randomized controlled trial. Aging Clinical and Experimental Research, 31(10), 1517–1524. 30539540 10.1007/s40520-018-1090-y

[eph13865-bib-0040] Karlsson, M. , Nilsson, J. A. , Sernbo, I. , Redlund‐Johnell, I. , Johnell, O. , & Obrant, K. J. (1996). Changes of bone mineral mass and soft tissue composition after hip fracture. Bone, 18(1), 19–22. 8717532 10.1016/8756-3282(95)00422-x

[eph13865-bib-0041] Kramer, I. F. , Blokhuis, T. J. , Verdijk, L. B. , van Loon, L. J. C. , & Poeze, M. (2019). Perioperative nutritional supplementation and skeletal muscle mass in older hip‐fracture patients. Nutrition Reviews, 77(4), 254–266. 30624706 10.1093/nutrit/nuy055

[eph13865-bib-0042] Lloyd, M. , Ademi, Z. , Harris, I. A. , Naylor, J. , Lewis, P. , de Steiger, R. , Buchbinder, R. , Wan, A. , & Ackerman, I. N. (2024). Implementing an enhanced recovery from surgery pathway to reduce hospital length of stay for primary hip or knee arthroplasty: A budget impact analysis. BMC Health Services Research, 24(1), 1540–1547. 39633364 10.1186/s12913-024-11871-7 PMC11616323

[eph13865-bib-0043] Long, H. , Liu, Q. , Yin, H. , Wang, K. , Diao, N. , Zhang, Y. , Lin, J. , & Guo, A. (2022). Prevalence trends of site‐specific osteoarthritis from 1990 to 2019: Findings from the global burden of disease study 2019. Arthritis & Rheumatology, 74(7), 1172–1183. 35233975 10.1002/art.42089 PMC9543105

[eph13865-bib-0044] Marshall, R. N. , Smeuninx, B. , Morgan, P. T. , & Breen, L. (2020). Nutritional strategies to offset disuse‐induced skeletal muscle atrophy and anabolic resistance in older adults: From whole‐foods to isolated ingredients. Nutrients, 12(5), 1533. 32466126 10.3390/nu12051533 PMC7284346

[eph13865-bib-0045] Matharu, G. S. , Culliford, D. J. , Blom, A. W. , & Judge, A. (2022). Projections for primary hip and knee replacement surgery up to the year 2060: An analysis based on data from the National Joint Registry for England, Wales, Northern Ireland and the Isle of Man. Annals of the Royal College of Surgeons of England, 104(6), 443–448. 34939832 10.1308/rcsann.2021.0206 PMC9157920

[eph13865-bib-0046] McKendry, J. , Coletta, G. , Nunes, E. A. , Lim, C. , & Phillips, S. M. (2024). Mitigating disuse‐induced skeletal muscle atrophy in ageing: Resistance exercise as a critical countermeasure. Experimental Physiology, 109(10), 1650–1662. 39106083 10.1113/EP091937 PMC11442788

[eph13865-bib-0047] Muyskens, J. B. , Foote, D. M. , Bigot, N. J. , Strycker, L. A. , Smolkowski, K. , Kirkpatrick, T. K. , Lantz, B. A. , Shah, S. N. , Mohler, C. G. , Jewett, B. A. , Owen, E. C. , & Dreyer, H. C. (2019). Cellular and morphological changes with EAA supplementation before and after total knee arthroplasty. Journal of Applied Physiology, 127(2), 531–545. 31343947 10.1152/japplphysiol.00869.2018 PMC6732445

[eph13865-bib-0048] Muyskens, J. B. , Winbush, A. , Foote, D. M. , Turnbull, D. W. , & Dreyer, H. C. (2020). Essential amino acid supplementation alters the p53 transcriptional response and cytokine gene expression following total knee arthroplasty. Journal of Applied Physiology, 129(4), 980–991. 32881622 10.1152/japplphysiol.00022.2020 PMC7654691

[eph13865-bib-0049] Ninomiya, K. , Takahira, N. , Ochiai, S. , Ikeda, T. , Suzuki, K. , Sato, R. , Ike, H. , & Hirakawa, K. (2020). Incidence of postoperative complications and non‐ periprosthetic fractures after total hip arthroplasty: A more than 10‐year follow‐up retrospective cohort study. Physical Therapy Research, 24(1), 77–83. 33981530 10.1298/ptr.E10043 PMC8111408

[eph13865-bib-0050] Nishizaki, K. , Ikegami, H. , Tanaka, Y. , Imai, R. , & Matsumura, H. (2015). Effects of supplementation with a combination of beta‐hydroxy‐beta‐methyl butyrate, L‐arginine, and L‐glutamine on postoperative recovery of quadriceps muscle strength after total knee arthroplasty. Asia Pacific Journal of Clinical Nutrition, 24(3), 412–420. 26420181 10.6133/apjcn.2015.24.3.01

[eph13865-bib-0051] Norte, G. E. , Knaus, K. R. , Kuenze, C. , Handsfield, G. G. , Meyer, C. H. , Blemker, S. S. , & Hart, J. M. (2018). MRI‐based assessment of lower‐extremity muscle volumes in patients before and after ACL reconstruction. Journal of Sport Rehabilitation, 27(3), 201–212. 28290752 10.1123/jsr.2016-0141

[eph13865-bib-0052] Nunes, E. A. , Stokes, T. , McKendry, J. , Currier, B. S. , & Phillips, S. M. (2022). Disuse‐induced skeletal muscle atrophy in disease and nondisease states in humans: Mechanisms, prevention, and recovery strategies. American Journal of Physiology. Cell Physiology, 322(6), C1068–C1084. 35476500 10.1152/ajpcell.00425.2021

[eph13865-bib-0053] Reider, L. , Owen, E. C. , Dreyer, H. C. , Fitton, L. S. , Willey, M. C. , & METRC (Major Extremity Trauma Research Consortium) . (2023). Loss of muscle mass and strength after hip fracture: An intervention target for nutrition supplementation. Current Osteoporosis Reports, 21(6), 710–718. 38019345 10.1007/s11914-023-00836-0

[eph13865-bib-0054] Ron, D. , Daley, A. B. , Coe, M. P. , Herrick, M. D. , Roth, R. H. , Abess, A. T. , Martinez‐Camblor, P. , Deiner, S. G. , & Boone, M. D. (2025). Frailty and associated healthcare expenditures among patients undergoing total hip and knee arthroplasty. The Journal of Frailty & Aging, 14(2), 100030. 40048426 10.1016/j.tjfa.2025.100030 PMC12184003

[eph13865-bib-0055] Schurch, M. A. , Rizzoli, R. , Slosman, D. , Vadas, L. , Vergnaud, P. , & Bonjour, J. P. (1998). Protein supplements increase serum insulin‐like growth factor‐I levels and attenuate proximal femur bone loss in patients with recent hip fracture. A randomized, double‐blind, placebo‐controlled trial. Annals of Internal Medicine, 128(10), 801–809. 9599191 10.7326/0003-4819-128-10-199805150-00002

[eph13865-bib-0056] Singh, J. A. , Mehta, B. , Mirza, S. Z. , Figgie, M. P. , Sculco, P. , Parks, M. , & Goodman, S. M. (2021). When has a knee or hip replacement failed? A patient perspective. Journal of Rheumatology, 48(3), 447–453. 31787606 10.3899/jrheum.191024

[eph13865-bib-0057] Singh, J. A. , Yu, S. , Chen, L. , & Cleveland, J. D. (2019). Rates of total joint replacement in the United States: Future projections to 2020–2040 using the National Inpatient Sample. Journal of Rheumatology, 46(9), 1134–1140. 30988126 10.3899/jrheum.170990

[eph13865-bib-0058] Tidermark, J. , Ponzer, S. , Carlsson, P. , Soderqvist, A. , Brismar, K. , Tengstrand, B. , & Cederholm, T. (2004). Effects of protein‐rich supplementation and nandrolone in lean elderly women with femoral neck fractures. Clinical Nutrition, 23(4), 587–596. 15297095 10.1016/j.clnu.2003.10.006

[eph13865-bib-0059] Tipton, K. D. (2010). Nutrition for acute exercise‐induced injuries. Annals of Nutrition & Metabolism, 57(Suppl. 2), 43–53. 21346336 10.1159/000322703

[eph13865-bib-0060] Van Koevering, M. , & Nissen, S. (1992). Oxidation of leucine and alpha‐ketoisocaproate to beta‐hydroxy‐beta‐methylbutyrate in vivo. American Journal of Physiology, 262(Pt 1), 27. 10.1152/ajpendo.1992.262.1.E27 1733247

[eph13865-bib-0061] Wainwright, T. W. , Gill, M. , McDonald, D. A. , Middleton, R. G. , Reed, M. , Sahota, O. , Yates, P. , & Ljungqvist, O. (2020). Consensus statement for perioperative care in total hip replacement and total knee replacement surgery: Enhanced Recovery After Surgery (ERAS((R))) Society recommendations. Acta Orthopaedica, 91(1), 3–19. 31663402 10.1080/17453674.2019.1683790 PMC7006728

[eph13865-bib-0062] Wainwright, T. W. , Immins, T. , & Middleton, R. G. (2024). Changes in hip and knee arthroplasty practice post‐COVID‐19 in the English NHS: A retrospective analysis of hospital episode statistics data. Annals of the Royal College of Surgeons of England, 107(4), 253–256. 39570318 10.1308/rcsann.2024.0100 PMC11957841

[eph13865-bib-0063] Wall, B. T. , Dirks, M. L. , & van Loon, L. J. C. (2013). Skeletal muscle atrophy during short‐term disuse: Implications for age‐related sarcopenia. Ageing Research Reviews, 12(4), 898–906. 23948422 10.1016/j.arr.2013.07.003

[eph13865-bib-0064] Wall, J. , Paul, M. , & Phillips, B. E. (2023). Nutritional interventions in prehabilitation for cancer surgery. Current Opinion in Clinical Nutrition and Metabolic Care, 26(6), 497–507. 37610824 10.1097/MCO.0000000000000974 PMC10552833

[eph13865-bib-0065] Weijzen, M. E. G. , Kouw, I. W. K. , Verschuren, A. A. J. , Muyters, R. , Geurts, J. A. , Emans, P. J. , Geerlings, P. , Verdijk, L. B. , & van Loon, L. J. C. (2019). Protein intake falls below 0.6 g*kg‐1*d‐1 in healthy, older patients admitted for elective hip or knee arthroplasty. Journal of Nutrition, Health and Aging, 23(3), 299–305. 10.1007/s12603-019-1157-2 PMC6399806 30820520

[eph13865-bib-0066] Weimann, A. , Braga, M. , Carli, F. , Higashiguchi, T. , Hubner, M. , Klek, S. , Laviano, A. , Ljungqvist, O. , Lobo, D. N. , Martindale, R. , Waitzberg, D. L. , Bischoff, S. C. , & Singer, P. (2017). ESPEN guideline: Clinical nutrition in surgery. Clinical Nutrition, 36(3), 623–650. 28385477 10.1016/j.clnu.2017.02.013

[eph13865-bib-0067] Wilkinson, D. J. , Hossain, T. , Hill, D. S. , Phillips, B. E. , Crossland, H. , Williams, J. , Loughna, P. , Churchward‐Venne, T. , Breen, L. , Phillips, S. M. , Etheridge, T. , Rathmacher, J. A. , Smith, K. , Szewczyk, N. J. , & Atherton, P. J. (2013). Effects of leucine and its metabolite beta‐hydroxy‐beta‐methylbutyrate on human skeletal muscle protein metabolism. The Journal of Physiology, 591(11), 2911–2923. 23551944 10.1113/jphysiol.2013.253203 PMC3690694

[eph13865-bib-0068] Wilkinson, D. J. , Hossain, T. , Limb, M. C. , Phillips, B. E. , Lund, J. , Williams, J. P. , Brook, M. S. , Cegielski, J. , Philp, A. , Ashcroft, S. , Rathmacher, J. A. , Szewczyk, N. J. , Smith, K. , & Atherton, P. J. (2018). Impact of the calcium form of beta‐hydroxy‐beta‐methylbutyrate upon human skeletal muscle protein metabolism. Clinical Nutrition, 37, (6 Pt A), 2068–2075. 29097038 10.1016/j.clnu.2017.09.024 PMC6295980

[eph13865-bib-0069] Wischmeyer, P. E. , Carli, F. , Evans, D. C. , Guilbert, S. , Kozar, R. , Pryor, A. , Thiele, R. H. , Everett, S. , Grocott, M. , Gan, T. J. , Shaw, A. D. , Thacker, J. K. M. , Miller, T. E. , Hedrick, T. L. , McEvoy, M. D. , Mythen, M. G. , Bergamaschi, R. , Gupta, R. , Holubar, S. D. , … Perioperative Quality Initiative (POQI) 2 Workgroup . (2018). American society for enhanced recovery and perioperative quality initiative joint consensus statement on nutrition screening and therapy within a surgical enhanced recovery pathway. Anesthesia and Analgesia, 126(6), 1883–1895. 29369092 10.1213/ANE.0000000000002743

[eph13865-bib-0070] Wu, Z. , Wang, Y. , Li, C. , Li, J. , Chen, W. , Ye, Z. , Zeng, Z. , Hong, K. , Zhu, Y. , Jiang, T. , Lu, Y. , Liu, W. , & Xu, X. (2022). Preoperative strength training for clinical outcomes before and after total knee arthroplasty: A systematic review and meta‐analysis. Frontiers in Surgery, 9, 879593. 35937597 10.3389/fsurg.2022.879593 PMC9349363

[eph13865-bib-0071] Yoshiko, A. , Beppu, M. , Izumida, R. , Matsubara, M. , Otani, T. , Shiratsuchi, H. , Takahira, N. , Moritani, T. , & Watanabe, K. (2020). Long‐term assessment of morphological, functional, and quantitative parameters of skeletal muscle in older patients after unilateral total hip arthroplasty. Experimental Gerontology, 137, 110971. 32422227 10.1016/j.exger.2020.110971

